# Radiofrequency Ablation for Treatment of Refractory Gastric Antral Vascular Ectasia: A Systematic Review of the Literature

**DOI:** 10.1155/2017/5609647

**Published:** 2017-08-01

**Authors:** M. Maida, S. Camilleri, M. Manganaro, S. Garufi, G. Scarpulla

**Affiliations:** Section of Gastroenterology, S. Elia-M. Raimondi Hospital, Caltanissetta, Italy

## Abstract

**Background and Study Aims:**

GAVE is an uncommon cause of upper nonvariceal bleeding and often manifests itself as occult bleeding with chronic anemia. To date, the standard of care for GAVE is endoscopic treatment with thermoablative techniques. Despite good technical results, approximately two thirds of patients remain dependent on transfusions after the therapy. One of the emerging and more promising endoscopic treatments for GAVE is radiofrequency ablation (RFA). The aim of this study is to perform a systematic review of literature in order to assess current evidence supporting the effectiveness of this technique for treatment of refractory GAVE.

**Materials and Methods:**

Through electronic search, we identified 14 records, and after removal of duplicates and irrelevant studies, we selected 10 studies on radiofrequency ablation of GAVE: 4 prospective open-label single-center studies, 1 retrospective multicentric study, and 5 case reports.

**Results:**

Among all 72 treated patients reported in literature, 74.3% achieved a clinical response, while nonfatal AEs have been reported in 4.2% of cases.

**Conclusions:**

Despite some qualitative limitations, all literature data support effectiveness of RFA for treatment of refractory GAVE. In the future, large prospective controlled trials with adequate follow-up are needed to better assess the effectiveness and safety of this procedure.

## 1. Introduction

Gastric antral vascular ectasia (GAVE) is a relatively uncommon disorder, which accounts for 4% of nonvariceal upper GI bleeding. The first description of the disease dates back to 1953 when Rider et al. described a case of an “erosive type of gastritis with marked venocapillary ectasia” in a patient with chronic iron deficiency anemia [1].

The typical endoscopic picture of GAVE has been better defined in 1984 by Jabbari et al. [2] who described the presence of “longitudinal antral folds converging on the pylorus, containing visible columns of tortuous red ectatic vessels.” Since endoscopic appearance of GAVE resembles the stripes of a watermelon, it is also known as the “watermelon stomach.” The second, less frequent, variant of GAVE is a “diffuse form,” in which red stripes are not visible and vascular ectasia presents itself with many angiodysplastic lesions in the antrum. In addition to its pathognomonic appearance, GAVE also presents sensitive, but not specific, histological features, namely, ectasia of mucosal capillaries, intravascular fibrin thrombosis, fibromuscular hyperplasia of the lamina propria, and spindle cell proliferation [3].

Diagnosis of GAVE is possible trough endoscopic detection of typical lesions and subsequent histological confirmation. Although diagnosis is generally easy, the therapeutic management of the disease is difficult and challenging. Most patients receive one or more treatments and require frequent blood transfusions, which result in a large number of hospital admissions and high costs.

Over time, several pharmacological, endoscopic, and surgical treatments have been proposed without fully satisfactory results [4–11].

The current standard of care for GAVE is endoscopic treatment with thermoablative techniques. Among these, one of the most widely used approaches is argon plasma coagulation (APC), a noncontact thermal method of hemostasis, which uses argon gas to deliver high-frequency current to the adjacent tissue [12–18]. Nevertheless, despite the initial effectiveness of endoscopic therapy, approximately two thirds of patients remain dependent on blood transfusions after treatment [19]. Beside this, also mechanical treatments such as endoscopic band ligation (EBL) have been proposed with promising results [20, 21].

One of the major emerging endoscopic treatments for GAVE is radiofrequency ablation (RFA) by the HALO system. This technology has been primarily developed and used for treatment of Barrett's esophagus with good results. Despite its original destination, the technique has also been used in the setting of lower gastrointestinal bleeding secondary to radiation proctitis [22, 23]. Since ectatic vessels secondary to chronic radiation proctitis present features similar to gastric antral vascular ectasia, RFA has also been tested in the treatment of GAVE as follows.

Focal catheters (Barrx™ HALO^90^ and HALO^ULTRA^) are installed on the distal end of the gastroscope. These devices present a bipolar electrode, with different surface sizes, and are provided with a flexible strap that slides over the tip.

Beside focal catheters, the Barrx through-the-scope (TTS) RFA catheter can be used. Unlike the former, this has a smaller surface and can be conveniently introduced trough the working channel, thus providing a greater maneuverability and avoiding multiple endoscope introductions (main specifications of devices are summarized in Table 1). After selection and setup of the catheter, the operator reaches the pathological gastric area and advances in order to allow the use of the device under direct vision. The catheter is placed against the target area at a 6 o'clock or 12 o'clock position, maintaining the big wheel of the endoscope, respectively, upwards or downwards to ensure maximum adherence with the tissue. Two to four consecutive pulses of energy can be delivered to the electrode in the same area (energy density of 12 J/cm^2^ and power density of 40 W/cm^2^) without moving the device or the electrode and keeping count of the total number of ablations. The electrode is therefore progressively repositioned to a new adjacent zone, starting at the pylorus and moving proximally, repeating the procedure until all the pathological gastric mucosa have been successfully treated. In order to maintain a correct energy delivery and to ensure full effectiveness in every area, the device needs to be removed and cleaned when the generator displays a reduced ablation percentage.

## 2. Materials and Methods

We performed a systematic review of the literature with the aim to summarize current evidences in support of radiofrequency ablation for GAVE and to better understand its effectiveness and its safety profile in this setting.

PubMed Central/Medline and Embase were searched systemically for English records up to December 2016 by two independent reviewers (M. M. and S. G.). Searches included combinations of the keywords “GAVE,” “gastric antral vascular ectasia,” or “antral vascular ectasia” with one or more of the following in both the title and/or the abstract: “thermal ablation,” “radiofrequency ablation,” “ablation,” “RFA,” “HALO,” or “Barrx”. Database searches were supplemented with literature searches of reference lists to find additional studies.

We considered eligible for the inclusion in this systematic review all original reports with the following characteristics: (1) inclusion of human subjects of any age treated with RFA for GAVE; (2) clear report of technical and clinical efficacy outcomes.

Studies investigating the effectiveness of other treatments for GAVE, alone or in combination with RFA, were excluded. Moreover, neither animal model studies nor nonoriginal reports (including reviews, systematic reviews, and meta-analysis) were included.

## 3. Results

Through electronic search strategies, 14 records have been identified, and after removal of duplicates and irrelevant studies, 10 studies have been finally selected: 4 prospective open-label single-center studies, 1 retrospective multicentric study, and 5 case reports (Table 2).

The first prospective open-label pilot study has been performed on 6 consecutive patients with GAVE, 4 of them refractory to APC, all treated by HALO^90^ ablation system for an average of 1.3 sessions per patient (ranging 1–3). All procedures resulted in a technical success with no adverse events, average hemoglobin increased from 8.6 g/dL to 10.2 g/dL after a 2-month follow-up period, and 5 out of 6 patients maintained stable hemoglobin levels and were no longer dependent on blood transfusions [24].

A subsequent open-label prospective study has been performed on 21 patients, all of them failures after a minimum of 2 APC sessions, and treated with HALO^90^ ULTRA catheter ablation system. Technical success was achieved in 19 out of 21 patients (90%), while clinical success, defined as independence from transfusions after a 6-month follow-up period and negative endoscopy 6 months after treatment, was achieved in 18 out of 21 patients (86%) with a mean hemoglobin increment from 8.6 g/dL to 10.2 g/dL. Only two adverse events occurred, one minor acute bleeding and one superficial ulceration, both resolved without medical intervention [25].

Other two prospective open-label studies, performed on patients with refractory GAVE treated with RFA, recently confirmed these results.

The first study, conducted on 7 patients treated with the HALO^ULTRA^ system, showed technical and clinical effectiveness in all patients with a mean gain of hemoglobin from 9.3 to 10.1 g/dL during a median follow-up of 6 months, in the absence of adverse events [26].

The second, performed on 9 patients treated with the HALO^90^ system, confirmed a technical success in 100% of the patients and a clinical success in 67% of the patients after an 11-month follow-up period, in the absence of complications [27].

Despite the good results, all these studies are single center and don't present a randomized design. In addition, most of them present short follow-up periods; therefore, the long-term effectiveness of the therapy is not known.

One recent retrospective multi-center open-label study performed in over 8 endoscopic centers in Europe and the USA, revised a series of 24 patients with GAVE who underwent thermoablation with the HALO system, 18 of them refractory to previous medical or endoscopic therapy and 23 with transfusion-dependent anemia. After a follow-up period of 6 months, all of them reduced their transfusional request, 15 out of 24 (65.2%) were no longer dependent on transfusions and an increase in hemoglobin levels (from 6.8 ± 1.4 to 9.8 ± 1.8 g/dL) was observed in all patients [28]. Like previous studies, this multicentric experience confirmed that RFA with HALO system is effective and safe for the treatment of GAVE. Similarly, other reports showed a good efficacy and safety of the procedure, in the absence of major side effects [29–32]. Only one case reported the occurrence of bacteremia and sepsis after RFA, which were resolved after antibiotic therapy [33].

## 4. Discussion

Despite recent technological advances, therapeutic management of GAVE is still challenging and presents many pitfalls.

Over time several pharmacological, endoscopic and surgical treatments have been proposed without satisfactory results. Medical therapy has yielded poor results, with high rates of adverse events [4–11], while surgical options, such as antrectomy, are curative but burdened by high morbidity and mortality and are no longer used in clinical practice.

The current standard of care is endoscopic treatment with thermoablative techniques. To date, some studies have proven the efficacy of APC in the treatment of GAVE, showing promising clinical outcomes with an improvement of anemia and a reduction of blood transfusion requests over time [12–18]. Nevertheless, most of these studies have been performed on small sample sizes with a short follow-up period, and few data exist on long-term outcomes.

Endoscopic band ligation (EBL) is already being widely used for the treatment of upper and lower vascular diseases. It is considered to be safer and cheaper than ablative techniques. A recent prospective study performed on 21 subjects with GAVE treated with EBL showed a clinical response in 91% of patients with a significant improvement in the mean hemoglobin levels and a significant decrease in blood transfusion requirements in the absence of major complications [20].

Despite good technical results of these techniques, a considerable proportion of patients relapses after endoscopic therapy and little is known regarding proper therapeutic strategies to apply for refractory GAVE.

Radiofrequency ablation with the Barrx system is a new, promising technique for endoscopic treatment of GAVE. The data synthesis deriving from this systematic review shows that, among all 72 treated patients reported in the literature, 74.3% achieved a clinical response, minor AEs occurred in 4.2% of cases, and no fatal AEs have been reported. Nevertheless, all these studies present some qualitative limitations. Most of them were performed on small samples, in a single center, and none of them were controlled. In addition, all were characterized by a short follow-up, making it difficult to assess the real rates of long-term clinical response after RFA.

Furthermore, a validated treatment schedule is absent. Some authors perform RFA with focal catheters, while others use through-the-scope (TTS) catheter. Moreover, in most centers, RFA is repeated every 4–8 weeks until there is evidence of complete endoscopic healing of the disease, while in other centers, the procedure is performed “on-demand” based on clinical response.

Best intervals, as well as catheter type and size, should ideally be identified on the basis of proper prospective or, alternatively, observational studies demonstrating a better performance of one or the other in achieving a technical and clinical success.

One further point must be made about costs. Considering a single session, RFA devices are more expensive than APC. On the other hand, it must be pointed out that RFA requires a lower number of sessions and, if effective, it could also reduce secondary costs related to repeated hospitalizations, endoscopic procedures, and blood transfusions.

## 5. Conclusions

Results from this systematic review suggest that radiofrequency ablation is a valuable therapeutic tool for treatment of refractory gastric antral vascular ectasia.

Current literature data support the effectiveness and safety of this procedure for treatment of GAVE, in the absence of major adverse events. Unfortunately, most of these evidences are burdened by qualitative limitations; therefore, in the future, larger prospective controlled trials with adequate follow-up are needed to better assess the clinical efficacy of the technique, as well as a validated treatment schedule. Finally, a cost analysis should be performed in order to estimate the cost effectiveness of radiofrequency ablation compared to other techniques currently in use.

## Figures and Tables

**Table 1:** Main technical specifications of RFA devices used for treatment of GAVE.

Devices	Electrode length	Electrode width	Catheter length	Maximum treatment per session
Channel catheter	15.7 mm	7.5 mm	135 cm	120
HALO^60^	15 mm	10 mm	160 cm	80
HALO^90^	20 mm	13 mm	160 cm	80
HALO^ULTRA^	40 mm	13 mm	160 cm	80

**Table 2:** Current available evidences on treatment of GAVE with radiofrequency ablation.

Author	Year	Study level	Sample	RFA system	Average sessions	Technical success	Clinical success	AEs	Follow-up (months)
Gross et al. [24]	2008	Prospective open-label single center	6	HALO^90^	Mean 1.7, range (1–3)	6/6 (100%)	5/6 (83%)	None	2
							Mean Hb gain 8.6 → 10.2		
McGorisk et al. [25]	2013	Prospective open-label single center	21	HALO^ULTRA^	Median 2, range (1–3)	19/21 (90%)	18/21 (86%)	2/21 (9.5%)	6
							Mean Hb gain 7.8 → 10.2		
Jana et al. [26]	2015	Prospective open-label single center	7	HALO^ULTRA^	Median 2, range (1–3)	7/7 (100%)	5/7 (71%)	None	6
							Mean Hb gain 9.3 → 10.1		
Raza and Diehl [27]	2015	Prospective open-label single center	9	HALO^90^	Median 3, range (2–6)	9/9 (100%)	6/9 (67%)	None	11
							Mean Hb gain 7.3 → 10.5		
Dray et al. [28]	2014	Retrospective multicentric	24	HALO^90^ or HALO^ULTRA^	Mean 1.8 ± 0.8	Not reported	15/24 (62%)	None	6
							Mean Hb gain 6.8 → 9.8		
Thandassery et al. [29]	2014	Case report	1	HALO^90^	2	Yes	Yes	None	1
							Hb gain 8 → 12		
Islam et al. [30]	2014	Case report	1	HALO^TTS^	1	Yes	Yes	None	1
							Hb gain 6→ 10.7		
Ibáñez-Sanz et al. [31]	2015	Case report	1	HALO^90^	2	Yes	Yes	None	3
							Hb gain 7.5 →12		
Trindade et al. [32]	2016	Case report	1	HALO^Nd^	3	Yes	Not reported	None	6
Gaslightwala et al. [33]	2014	Case report	1	HALO^90^	4	Not reported	Not reported	Yes	Not reported

Technical success: feasibility of therapy and complete endoscopic ablation of GAVE; clinical success: hemoglobin improvement and complete independence from the need for transfusions after follow-up; RFA: radiofrequency ablation; AEs: adverse events.
